# Right Ventricular Volume and Function Assessment in Congenital Heart Disease Using CMR Compressed-Sensing Real-Time Cine Imaging

**DOI:** 10.3390/jcm10091930

**Published:** 2021-04-29

**Authors:** Benjamin Longère, Julien Pagniez, Augustin Coisne, Hedi Farah, Michaela Schmidt, Christoph Forman, Valentina Silvestri, Arianna Simeone, Christos V Gkizas, Justin Hennicaux, Emma Cheasty, Solenn Toupin, David Montaigne, François Pontana

**Affiliations:** 1 University of Lille, Inserm, CHU Lille, Institut Pasteur Lille, U1011—European Genomic Institute for Diabetes (EGID), F-59000 Lille, France; augustin.coisne@chru-lille.fr (A.C.); david.montaigne@chru-lille.fr (D.M.); francois.pontana@chru-lille.fr (F.P.); 2 CHU Lille, Department of Cardiovascular Radiology, F-59000 Lille, France; julien.pagniez@chru-lille.fr (J.P.); hedi.farah@chru-lille.fr (H.F.); valentina.silvestri@chru-lille.fr (V.S.); arianna.simeone@chru-lille.fr (A.S.); chgkizas@gmail.com (C.V.G.); justin.hennicaux@chru-lille.fr (J.H.); 3 MR Product Innovation and Definition, Magnetic Resonance, Siemens Healthcare GmbH, 91052 Erlangen, Germany; michaela.schmidt@siemens-healthineers.com (M.S.); christoph.forman@siemens-healthineers.com (C.F.); 4 Department of Cardiovascular Imaging, St Bartholomew’s Hospital, West Smithfield, London EC1A 7BE, UK; emma.cheasty@nhs.net; 5 Scientific Partnerships, Siemens Healthcare France, 93200 Saint-Denis, France; solenn.toupin@siemens-healthineers.com

**Keywords:** cardiac, heart, magnetic resonance, CMR, compressed sensing, congenital heart disease, GUCH, real-time imaging

## Abstract

Background and objective: To evaluate the reliability of compressed-sensing (CS) real-time single-breath-hold cine imaging for quantification of right ventricular (RV) function and volumes in congenital heart disease (CHD) patients in comparison with the standard multi-breath-hold technique. Methods: Sixty-one consecutive CHD patients (mean age = 22.2 ± 9.0 (SD) years) were prospectively evaluated during either the initial work-up or after repair. For each patient, two series of cine images were acquired: first, the reference segmented multi-breath-hold steady-state free-precession sequence (SSFP_ref_), including a short-axis stack, one four-chamber slice, and one long-axis slice; then, an additional real-time compressed-sensing single-breath-hold sequence (CS_rt_) providing the same slices. Two radiologists independently assessed the image quality and RV volumes for both techniques, which were compared using the Wilcoxon test and paired Student’s *t* test, Bland–Altman, and linear regression analyses. The visualization of wall-motion disorders and tricuspid-regurgitation-related signal voids were also analyzed. Results: The mean acquisition time for CS_rt_ was 22.4 ± 6.2 (SD) s (95% CI: 20.8–23.9 s) versus 442.2 ± 89.9 (SD) s (95% CI: 419.2–465.2 s) for SSFP_ref_ (*p* < 0.001). The image quality of CS_rt_ was diagnostic in all examinations and was mostly rated as good (*n* = 49/61; 80.3%). There was a high correlation between SSFP_ref_ and CS_rt_ images regarding RV ejection fraction (49.8 ± 7.8 (SD)% (95% CI: 47.8–51.8%) versus 48.7 ± 8.6 (SD)% (95% CI: 46.5–50.9%), respectively; *r* = 0.94) and RV end-diastolic volume (192.9 ± 60.1 (SD) mL (95% CI: 177.5–208.3 mL) versus 194.9 ± 62.1 (SD) mL (95% CI: 179.0–210.8 mL), respectively; *r* = 0.98). In CS_rt_ images, tricuspid-regurgitation and wall-motion disorder visualization was good (area under receiver operating characteristic curve (AUC) = 0.87) and excellent (AUC = 1), respectively. Conclusions: Compressed-sensing real-time cine imaging enables, in one breath hold, an accurate assessment of RV function and volumes in CHD patients in comparison with standard SSFP_ref_, allowing a substantial improvement in time efficiency.

## 1. Introduction

The advent of heart surgery and percutaneous cardiac procedures has considerably improved outcomes in patients born with congenital heart disease [1]. It has led to a growing number of adult survivors with complex congenital heart diseases, with a concomitantly increasing need for imaging follow-up in this clinical context.

Right ventricular (RV) function and volume assessment is of paramount importance in many of these patients, such as in post-repair tetralogy of the Fallot population, as treatment decisions and outcomes mainly rely on RV parameters according to the European Society of Cardiology guidelines for the management of adult congenital heart disease [2]. Although echocardiography remains the first-line investigation, cardiac magnetic resonance (CMR) is a method of choice for RV morphological and functional evaluation in congenital heart disease due to its complex geometry. CMR is considered superior to echocardiography for the evaluation of RV and should be regularly used when the information is essential for patient management, i.e., for quantification of RV volume and ejection fraction, quantification of pulmonary regurgitation, evaluation of RV outflow-tract and pulmonary arteries, detection of myocardial fibrosis or scar, and tissue characterization [2].

However, one major limitation of such extensive CMR examinations is currently the acquisition time, which can be difficult to tolerate in this population, as well as the iterative breath holds, which can be difficult to maintain, leading to poor-quality examinations because of breathing artifacts. To reduce this limitation, the development of acceleration techniques in MR imaging is crucial, and compressed sensing (CS) represents a promising technique in this category. Schematically, CS is a technique that combines a strong and random *k*-space subsampling, thus enabling a very high scan speed, and it uses non-linear iterative reconstructions to make the final image look as close as possible to that if the *k*-space had been fully sampled. The use of CS for CMR cine imaging theoretically enables real-time acquisition with whole-ventricle coverage in a single breath hold, and its reliability has been successfully tested in previous studies for left ventricular (LV) or sometimes right ventricular (RV) functional assessment in healthy volunteers and in patients with various extra-congenital pathologies [3,4,5,6,7,8,9,10,11].

We thus aimed at evaluating the reliability of real-time cine imaging using the CS technique for quantification of RV and LV function and volumes in congenital heart disease patients in comparison with conventional multi-breath-hold segmented steady-state free-precession cine imaging.

## 2. Materials and Methods

### 2.1. Study Population

From January to April 2019, 61 consecutive patients were prospectively included. All patients were clinically scheduled for CMR in the context of congenital heart disease for either the initial work-up or after repair. A single-ventricle anatomy was considered as an exclusion criterion. The protocol was approved by our institutional Ethics Committee, and the patients gave informed consent.

### 2.2. CMR Protocol

All CMR examinations were performed on a 1.5 T magnetic resonance scanner (MAGNETOM Aera, Siemens Healthcare, Erlangen, Germany). For each patient, two series of two-dimensional cine images were systematically acquired: prospectively triggered segmented multi-breath-hold steady-state free-precession sequence (SSFP_ref_) was considered as the reference technique, including a conventional short-axis stack, one LV and one RV two-chamber slice, and one four-chamber slice with an 8 mm slice thickness and a 2 mm gap; an additional prospectively triggered real-time CS sequence (CS_rt_) in a single breath hold. CS_rt_ cine images were acquired with the same slice number, position, and thickness as those used in the reference technique. An additional phase-contrast imaging sequence was acquired on the pulmonary trunk to assess the RV stroke volume and the severity of the encountered tricuspid regurgitation. The details of the imaging parameters are listed in Table 1.

### 2.3. Functional Evaluation

The quantitative assessment consisted of the evaluation of RV functional parameters with both the SSFP_ref_ and CS_rt_ sequences, i.e., ejection fraction (EF), end-diastolic volume (EDV), end-systolic volume (ESV), and stroke volume (SV). The same parameters were measured for the left ventricle, as well as the LV mass. For these quantitative measurements, endocardial and epicardial contours were segmented on the conventional short-axis stacks of cine images using a dedicated analysis software (Cardiac MR analysis workflow, Syngo.via VB30A, Siemens Healthcare, Erlangen, Germany). According to our CMR practice, RV trabeculations were included in the RV volume. Four-chamber and long-axis slices were used as reference images to trace the atrio-ventricular valve planes to ensure an optimal delineation of the heart base for an accurate volume calculation.

### 2.4. Image Quality Assessment

The overall subjective image quality of the SSFP_ref_ and CS_rt_ cine images was rated on the basis of a four-point Likert scale as follows: 4 = excellent, 3 = good, 2 = fair image quality, and 1 = non-diagnostic examination.

In addition, the objective RV image quality was assessed using previously published criteria, which are mostly based on artifact rating, and they were adapted to the RV [12]. Schematically, 1 point was given if an artifact (fold-over, respiratory ghost, cardiac ghost, image blurring/mistriggering, metallic, or shimming) hampered the visualization of the RV border at the end-systole and/or end-diastole; if such an artifact involved 2 or ≥3 slices, 2 or 3 points were given, respectively.

The depictions of the regional RV wall-motion abnormalities (i.e., hypokinetic, akinetic, or dyskinetic wall) were also rated at 4 anatomical levels (base, mid-cavity, apex, and RV outflow tract), and the depictions of tricuspid-regurgitation-related flow artifacts were assessed on the four-chamber slice.

### 2.5. Conditions of Image Analysis

The acquired SSFP_ref_ and CS_rt_ cine images were independently analyzed offline by a CMR radiologist (HF) with 3 years of experience. After anonymization, the images from both sequences were randomized and mixed. The two types of cine sequences from one patient were not read in the same session. A radiologist (JP) with 10 years of experience performed the functional RV assessment on a 20-patient sample for the determination of interobserver agreement with the new CS cine technique [13].

### 2.6. Statistical Analysis

Categorical data are represented as numbers (percentages). Continuous variables are represented as mean ± standard deviation (SD) (95% confidence interval (CI)) in the case of normal distribution and median (range: minimum–maximum) in other cases. SSFP_ref_ and CS_rt_ were compared using paired Student’s *t* test, Bland–Altman, and linear regression analyses. The interobserver agreement of CS_rt_ was determined by calculating the intra-class correlation coefficient. An analysis of variance was performed to compare the RV stroke volumes assessed with both cine sequences with the forward pulmonary volume assessed with PCI. Differences in quality scores between SSFP_ref_ and CS_rt_ were assessed using the Wilcoxon test. Values of *p* < 0.05 were considered statistically significant. For the depictions of valvular regurgitations and wall-motion disorders, a receiver operating characteristic (ROC) curve was used. The statistical analysis was performed with dedicated software (MedCalc 18.11, MedCalc Software bvba, Ostend, Belgium).

## 3. Results

### 3.1. Population Description

The 61 patients (29 men, 32 women; mean age: 22.2 ± 9.0 (SD) years; 95% CI: 19.9–24.5 years) underwent CMR for: tetralogy of Fallot (*n* = 33/61; 54.1%), pulmonary atresia with a ventricular septal defect (*n* = 7/61; 11.5%), cardiac shunt (*n* = 7/61; 11.5%), transposition of great arteries (*n* = 3/61; 4.9%), aortic coarctation (*n* = 2/61; 3.3%), congenital pulmonary stenosis (*n* = 2/61; 3.3%), cor triatriatum sinister (*n* = 2/61; 3.3%), congenitally corrected transposition of the great arteries (*n* = 2/61; 3.3%), pulmonary atresia with intact ventricular septum after biventricular repair (*n* = 2/61; 3.3%), and congenital aortic stenosis (*n* = 1/61; 1.6%). Table 2 summarizes further details of the characteristics of the study population.

### 3.2. Cine Acquisitions

The mean duration for single-breath-hold CS_rt_ acquisition was 22.4 ± 6.2 (SD) s (95% CI: 20.8–23.9 s) versus 442.2 ± 89.9 (SD) s (95% CI: 419.2–465.2 s) for SSFP_ref_ (*p* < 0.001). The mean acceleration factor provided by CS_rt_ was 20.8 ± 5.6 (95% CI: 19.3–22.2) as compared with SSFP_ref_. A mean number of 13.3 ± 2.9 slices (95% CI: 12.5–14.1 slices) was acquired with each sequence.

### 3.3. Quantitative Evaluation

**Figure 1:**
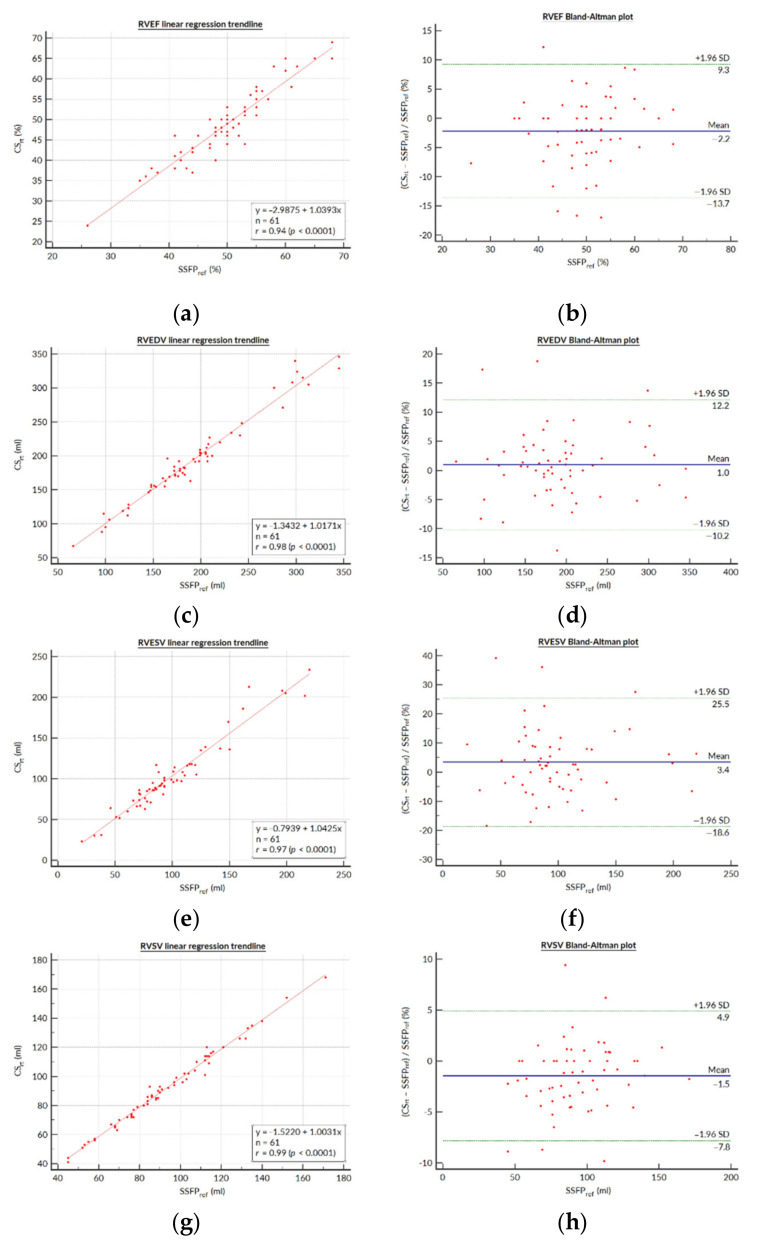
Bland–Altman plots and linear regression trendlines for quantification of the right ventricular functional parameters. Left column: Linear regression trend lines for (**a**) RVEF, (**c**) RVEDV, (**e**) RVESV, and (**g**) RVSV, representing the correlation between parameters measured on the SSFP_ref_ and CS_rg_ sequences. Right column: Bland–Altman plots for the (**b**) RVEF, (**d**) RVEDV, (**f**) RVESV, and (**h**) RVSV. Solid blue lines are the mean differences and dashed green lines are the 95% limits of agreement. Abbreviations: SSFP_ref_, reference steady-state free-precession cine; CS_rt_, real-time compressed-sensing cine; SD, standard deviation; RVEF, right ventricular ejection fraction; RVEDV, right ventricular end-diastolic volume; RVESV, right ventricular end-systolic volume; RVSV, right ventricular stroke volume.

Detailed results regarding the SSFP_ref_ and CS_rt_ segmentations for the RV and LV functional parameters are presented in Table 3. There was no statistically significant difference between mean SSFP_ref_ and CS_rt_ for RVEDV (192.9 ± 60.1 (SD) mL (95% CI: 177.5–208.3 mL) versus 194.9 ± 62.1 (SD) mL (95% CI: 179.0–210.8 mL), respectively; *p* = 0.169). The RVEF was slightly underestimated in the CS_rt_ images (CS_rt_: 48.7 ± 8.6 (SD) % (95% CI: 46.5–50.9%); SSFP_ref_: 49.8 ± 7.8 (SD) % (95% CI: 47.8–51.8%); *p* = 0.006) as a result of a statistically significant but not clinically relevant underestimation of the RVESV in CS_rt_. The analysis of variance did not demonstrate any significant differences with respect to the RV stroke volume, regardless of the measurement method (SSFP_ref_: 93.6 ± 25.7 (SD) mL (95% CI: 87.0–100.2 mL); CS_rt_: 92.3 ± 26.0 (SD) mL (95% CI: 85.7–99.0 mL); PCI: 88.6 ± 27.1 (SD) mL (95% CI: 91.6–95.5 mL); *p* = 0.605). No statistically significant differences were visible between SSFP_ref_ and CS_rt_ for LVEF and LVEDV. The LV mass was slightly overestimated in CS_rt_. The linear regression yielded good agreement between both acquisition techniques for all RV functional parameters (Figure 1), and the *r* values were excellent for all parameters. On the other hand, graphical analysis of the Bland–Altman plot demonstrated up to five (tetralogy of Fallot, *n* = 5/5; 100%) paired measurements out of the limits of agreement (LOA) depending on the RV parameter considered (LOA in RVEF bias: −13.7 to +9.3%).

### 3.4. Qualitative Evaluation

**Figure 2:**
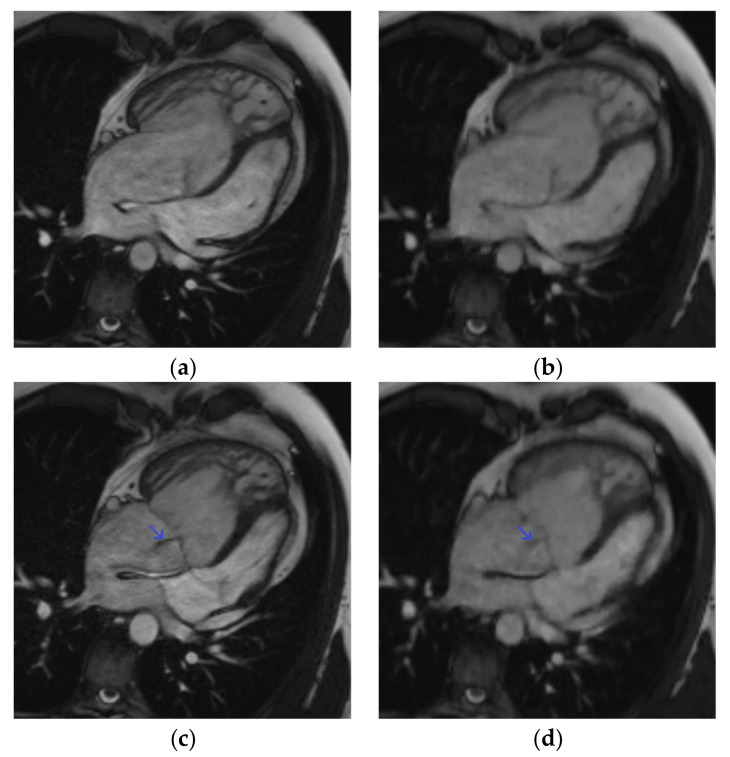
Four-chamber cine slice acquired with both sequences in a 31-year-old male patient referred for transposition of great arteries after a Senning repair follow-up. SSFP_ref_ view in the diastole (**a**) and systole (**c**); overall image quality score = 4/4; RVEF = 42%; EDV = 345 mL. The same slices acquired with CS_rt_ in the diastole (**b**) and systole (**d**); overall image quality score = 3/4; RVEF = 40%; EDV = 346 mL. The tricuspid regurgitation flow artifact remains conspicuous with both sequences (blue arrow). Abbreviations: SSFP_ref_, reference steady-state free-precession cine; CS_rt_, real-time compressed-sensing cine; RVEF, right ventricular ejection fraction; EDV, end-diastolic volume.

**Figure 3:**
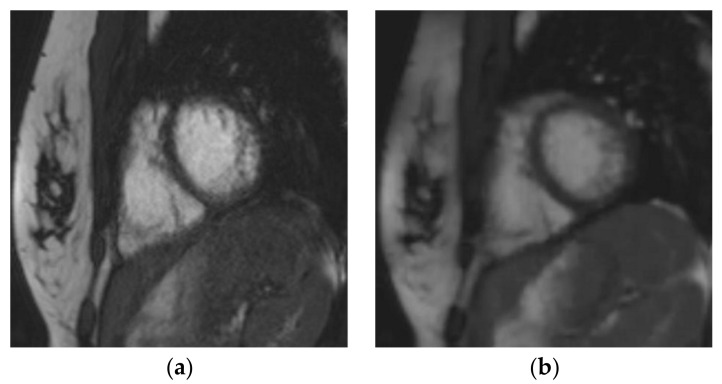

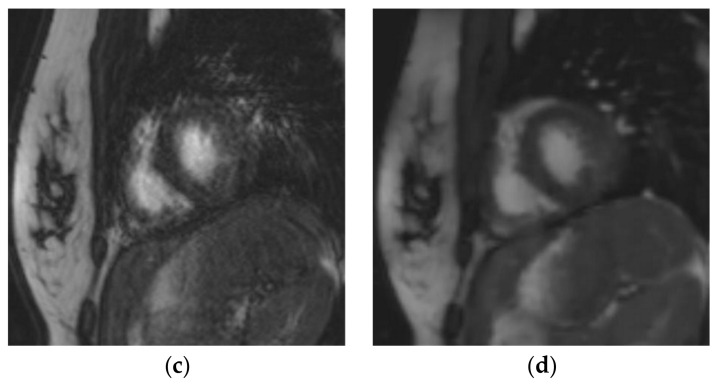
Short-axis cine slice acquired with both sequences in a 26-year-old female patient referred for a tetralogy of Fallot post-repair follow-up, demonstrating an irregular heart rate. Mean heart rate = 78 ± 14 (SD) bpm (range: 51 to 107 bpm). SSFP_ref_ view in the diastole (**a**) and systole (**c**); overall image quality score = 2/4; RVEF = 51%; EDV = 148 mL. The same slices were acquired with CS_rt_ in the diastole (**b**) and systole (**d**); overall image quality score = 3/4; RVEF = 48%; EDV = 154 mL. The fair image quality is due to the mis-triggering of artifacts in SSFP_ref_, while CS_rt_ provided both accurate segmentation and good image quality. Abbreviations: bpm, beats per minute; SD, standard deviation; SSFP_ref_, reference steady-state free-precession cine; CS_rt_, real-time compressed-sensing cine; RVEF, right ventricular ejection fraction; EDV, end-diastolic volume.

Figure 2; Figure 3; Video S1; Video S2 (Supplementary Materials) provide representative examples of the image quality achieved with CS_rt_ images in various clinical situations. The image quality of CS_rt_ was diagnostic in all examinations (Table 4). There was a significantly lower overall image quality score for CS_rt_ images (*p* = 0.0001) because most of the examinations were rated as excellent with SSFP_ref_ and good with CS_rt_. However, qualitative artifact presence was statistically lower in the CS_rt_ images than in SSFP_ref_ (*p* = 0.0016). Considering SSFP_ref_ as the gold standard, there were no diagnostic losses for regional RV wall-motion abnormalities in CS_rt_ images, demonstrating a 100% sensitivity and specificity (normokinetic: *n* = 157/244 (64.3%); hypokinetic: *n* = 39/244 (16.0%); akinetic: *n* = 1/244 (0.4%); dyskinetic: *n* = 47/244 (19.3%)). The tricuspid-regurgitation-flow void depictions in CS_rt_ images had a sensitivity and specificity of 74.2% and 100%, respectively (predictive positive value = 100%; predictive negative value = 78.9%; area under ROC = 0.87). Using SSFP_ref_, 23/61 (37.7%) tricuspid regurgitations were depicted (mild: 20/23 (87.0%); moderate: 3/23 (13.0%)). Of the 8/61 (13.1%) tricuspid regurgitations that were not depicted with the CS_rt_ cine, all were quantified as mild with the reference technique (the difference between RVSV and anterograde pulmonary volume was measured with the phase-contrast sequence) [14].

## 4. Discussion

Our prospective monocentric study based on a cohort of 61 pediatric and grown-up CHD patients, including 33 tetralogies of Fallot, demonstrated that the quantification of RV function and volumes yields similar results for CS_rt_ and for the standard SSFP_ref_ cine techniques, while the former allows a drastically shorter acquisition time. The agreement between CS_rt_ and SSFP_ref_ regarding the RV volume assessment is in line with the findings of previous studies performed on smaller cohorts of healthy volunteers and non-CHD patients [5,10]. The *t* test comparisons performed in our study demonstrated a statistically significant trend towards a 1.07% RVEF underestimation (relative mean difference = −2.14%), a 3.51 mL RVESV overestimation (relative mean difference = 3.54%), and a 1.28 mL RVSV underestimation (relative mean difference = −1.36%) with CS_rt_. The segmented steady-state free precession cine is currently considered the gold-standard technique for the measurement of ventricular volumes, including in CHD patients [2,15,16,17]. However, these trends must be balanced with the clinically relevant LOA demonstrated in the Bland–Altman plots, mainly regarding the relative bias of RVEF (−13.7; +9.3%), which was also the case in other studies evaluating CS cine for RV assessment in non-CHD populations, where the LOA was reported to be from −10.5 to +11.6% [5,10,18,19]. A more recent study also found a similar performance of a novel real-time steady-state free precession spiral sequence reconstructed using CS in a pediatric-only CHD population, but did not evaluate the regional wall-motion abnormalities or the depiction of tricuspid-regurgitation-related flow artifacts [19]. It must also be highlighted that both intra- and interobserver agreement was excellent for all CS_rt_-evaluated RV functional parameters. Additionally, our findings demonstrate a strong agreement between both SSFP_ref_ and CS_rt_ for functional LV parameters, despite the slight LV mass overestimation with CS_rt_, as previously reported [5].

The best image plane required for post-processing of RV volumes has long been debated, especially in the clinical context of CHD. However, it has been shown that despite a trend favoring the axial plane rather than the short axis in terms of reproducibility, there were no clinically significant differences between these two contouring methods [20]. Thus, we drew RV endocardial contours on short-axis stacks, as this is widely performed and is easier to set up in routine practice, but care was taken to trace the tricuspid valve on reference four-chamber and RV long-axis slices to delimit the right ventricular basis as precisely as possible. Despite controversies about segmentation methods, we included trabeculations in RV volumes according to our CMR center’s habits [21]. The justification for this choice lies in the need for consistency in our practice in order to preserve reproducibility in patient follow-ups [22,23]. Nevertheless, we acknowledge that excluding trabeculations from the blood volume could be more accurate [24]. This could explain the lower dispersion of the differences in parameters measured with SSFP_ref_ and CS_rt_ on Bland–Altman plots when the RVEF or RVESV increase (Figure 1b,h).

The CS_rt_ images were diagnostic in all examinations, but the overall image quality score was, as expected, significantly lower with this technique. This can be explained by the lower edge definition provided by CS, which resulted in a slightly blurry aspect of the images. This should be addressed by using a two-shot variant of the evaluated compressed-sensing cine, which would provide an improved edge sharpness and would preserve the important scan time reduction [25]. Nevertheless, this two-shot variant has not yet been evaluated for the right ventricular functional parameters and should be the subject of further study. However, a lower RV artifact score was found with CS_rt_ due to the reduction of artifacts—which were mostly related to mis-triggering—achieved with this real-time acquisition technique. In addition, the performance of CS_rt_ for RV wall-motion disorder depiction was very high, as there was no diagnostic loss in comparison with the reference images, and only mild tricuspid regurgitations were not depicted with CS_rt_ cine (Figure 2; Video S1 (Supplementary Materials)). These findings are in line with a recently published study evaluating the same real-time CS cine sequences for both LV and RV assessment in a non-selected adult cohort [18].

The CS_rt_ sequence consisted of a single-breath-hold cine acquisition; however, some patients could not fully achieve the required apnea due to their clinical condition. They were not excluded from the study, as our aim was to be as representative as possible of our CHD population that we encounter in daily practice. Despite these free-breathing ends of acquisition, no major artifacts (CMR RV quality score > 7/10) were noticed in the CS_rt_ images, which all had diagnostic quality. These findings strongly suggest the possibility of the free-breathing acquisition of CS_rt_ cine, which is particularly relevant for pediatric or end-stage CHD patients. Although the aim of this study was not to evaluate free-breathing imaging protocol, free-breathing CS_rt_ has been demonstrated to be a reliable alternative that allows faster acquisition than sequences based on registration of multiple acquisitions and motion-correction algorithms [9,26]. The acceleration provided by CS_rt_ may allow one to either (a) shorten breath-holding duration by splitting the stacks of cine slices to reduce the number of cine loops acquired per breath hold, especially for patients with shortness of breath, or (b) to shorten the overall examination duration, as suggested in the present study (average: 22 s (CS_rt_) versus 7 min and 22 s (SSFP_ref_) for 13 cine slices) to improve the clinical workflow and tolerance in children or to spare examination time in order to acquire additional sequences. Indeed, a comprehensive study of hemodynamic patterns is a key point in the initial work-up of CHD or in repair follow-up. Four-dimensional (4D) flow is a promising but time-consuming technique that may take advantage of the thusly spared time [27,28]. Depending on the sequence design, this technique may provide both qualitative and quantitative assessments of flow patterns and ventricular volumes [29]. As extended examination durations are an obstacle for the routine use of 4D flow, compressed-sensing 4D flow prototypes are also being developed [30,31,32].

Another interesting point is the decrease in the mis-triggering artifacts observed with CS_rt_ (Figure 3; Video S2 (Supplementary Materials)). Even though it was not the purpose of our study and would require further dedicated studies, this finding suggests that CS_rt_ might have an important part to play for functional or WMD evaluations in patients with irregular heart rates [33].

### Limitations

The minimum age in our population was 7 years, and further studies would be needed for validation in younger children. We also have to report that despite the drastic decrease in acquisition time, the data reconstruction process was more time consuming than with SSFP_ref_, as 2 min were necessary in order to visualize the whole cine stack in spite of a graphics processing unit upgrade.

Regarding the blurry aspect of images that we observed with CS, it must be said that the CS_rt_ sequence was designed in order to reduce the acquisition time as much as possible. In a different way, some authors have successfully tested CS cine to improve the spatial or temporal resolution with quite similar or even moderately shorter acquisition times than those for reference SSFP_ref_, or even to achieve three-dimensional cine acquisitions [34,35,36]. Our scan time was, however, strongly reduced, as low as 22.4 ± 6.2 (SD) s versus about 6 to 10 min for segmented multi-breath-hold SSFP_ref_, which can be very useful for patient comfort and workflow.

Although it is in line with the current literature, the bias observed between the two sequences in RVEF measurement (∼10%) is clinically relevant and must be taken into consideration in CHD follow-up [37,38]. The reason for such a bias may lie in the edge sharpness impairment induced by CS_rt_ as compared to SSFP_ref_ [18]. Indeed, the partial Fourier and the interpolation performed to provide a constant cardiac frame rate for post-processing induced a smoother and blurrier endocardial delineation than conventional cine. This limitation should be responsible for the increased bias in segmentation between the two techniques and may be solved by a multi-shot approach to CS acceleration [25].

## 5. Conclusions

Compressed-sensing real-time cine imaging enables the assessment RV function and volumes in patients with CHD while providing a significant reduction in examination duration and allowing an improvement in time efficiency and patient care.

## Figures and Tables

**Table 1:** Imaging parameters of the reference steady-state free-precession cine imaging and real-time compressed-sensing cine imaging.

Parameters	SSFP_ref_	CS_rt_
Repetition time—ms	3.16	2.70
Echo time—ms	1.23	1.14
Flip angle—degrees	57	60
Field of view—mm^2^	375 × 280	360 × 270
Matrix—pixels^2^	288 × 216	224 × 168
Spatial resolution—mm^2^	1.3 × 1.3	1.6 × 1.6
Temporal resolution—ms	41.2	49
Slice thickness/gap—mm	8/2	8/2
Bandwidth—Hz/pixel	915	900
ECG mode	Prospective triggering	Prospective triggering
Number of measured cardiac phases per cycle	20 ^a^	16 ± 4.1
Reconstructed cardiac frames per cycle—*n*	20 ^a^	20 ^b^
Number of views per frame—*n*	13.0 ± 3.7 ^c^	18 ^a^
Number of breath holds	13.3 ± 2.9	1 ^a^
Cycles of iterative reconstruction—*n*	NA	40
Breath-hold duration—cardiac cycle per slice	7	2 ^d^
Acceleration factor	2	11

Data are expressed as mean ± standard deviation in the absence of any indication. ^a^ Constant value. ^b^ Interpolation was performed to provide a constant frame rate of 20 cardiac phases per cycle for post-processing. ^c^ The number of views per frame was set according to the shorter R–R interval in order to acquire 20 cardiac phases. ^d^ The first cardiac cycle is required for signal preparation and the second one for signal acquisition. Abbreviations: SSFP_ref_, reference steady-state free-precession cine; CS_rt_, real-time compressed-sensing cine; ECG, electrocardiogram; *n*, data represented as numbers; NA, not applicable.

**Table 2:** Study population characteristics.

	Mean ± SD (95% CI)	Minimum Value	Maximum Value
Age—years	22.0 ± 9.0 (19.9–24.5)	7	53
Weight—kg	59.1 ± 16.8 (54.8–63.4)	24	100
Height—cm	163.4 ± 15.0 (159.5–167.2)	121	190
Body surface area—m^2^	1.6 ± 0.3 (1.6–1.7)	0.9	2.3
Heart rate—beats per minute	74.6 ± 14.2 (71.0–78.2)	44	112

Abbreviations: SD, standard deviation; 95% CI, 95% confidence interval.

**Table 3:** Functional parameters segmented on both the reference steady-state free-precession and real-time compressed-sensing cine.

	SSFP_ref_ Sequence (mean ± SD (95% CI))	CS_rt_ Sequence (mean ± SD (95% CI))	Mean Difference ± SD (95% CI)	Paired *t* Test *p*	ICC	
					Inter	Intra
RVEF—%	49.8 ± 7.8 (47.8–51.8)	48.7 ± 8.6 (46.5–50.9)	−1.07 ± 2.90 (−1.81 to −0.32)	0.006	0.95	0.94
RVEDV—mL	192.9 ± 60.1 (177.5–208.3)	194.9 ± 62.1 (179.0–210.8)	2.00 ± 11.21 (−0.87 to 4.87)	0.169	0.91	0.97
RVESV—mL	98.9 ± 41.0 (88.4–109.4)	102.4 ± 44.0 (91.1–113.7)	3.51 ± 11.05 (0.68–6.34)	0.016	0.97	0.98
RVSV—mL	93.6 ± 25.7 (87.0–100.2)	92.3 ± 26.0 (85.7–99.0)	−1.28 ± 2.96 (−2.04 to −0.52)	0.001	0.99	0.93
LVEF—%	57.4 ± 7.5 (55.4–59.3)	57.8 ± 7.9 (55.7–59.8)	0.38 ± 4.22 (−0.70 to 1.46)	0.488	0.98	0.98
LVEDV—mL	130.0 ± 40.1 (119.8–140.3)	128.7 ± 43.6 (117.5–139.8)	−1.39 ± 10.68 (−4.13 to 1.34)	0.312	0.98	0.97
LVESV—mL	56.3 ± 23.5 (50.3–62.3)	55.5 ± 27.1 (48.5–62.4)	−0.84 ± 10.24 (−3.46 to 1.79)	0.526	0.97	0.98
LVSV—mL	73.6 ± 21.9 (68.0–79.2)	73.4 ± 22.1 (67.7–79.0)	−0.23 ± 3.14 (−1.03 to 0.58)	0.571	0.99	0.99
LVM—g	95.7 ± 33.9 (87.0–104.4)	102.9 ± 38.5 (93.0–112.8)	7.18 ± 15.12 (3.31 to 11.05)	0.0005	0.96	0.97

ICC was used to evaluate the interobserver agreement for the RV segmentation. The significance of Student’s *t* test is defined by *p* < 0.05. Abbreviations: SSFP_ref_, reference steady-state free-precession cine; CS_rt_, real-time compressed-sensing cine; SD, standard deviation; 95% CI, 95% confidence interval; RV, right ventricular; LV, left ventricular; EDV, end-diastolic volume; ESV, end-systolic volume; SV, stroke volume; LVM, left ventricular mass; ICC, intraclass correlation coefficient; Inter, interobserver; Intra, intraobserver.

**Table 4:** Qualitative assessment of the reference steady-state free-precession cine and real-time compressed-sensing cine.

**a.** Image quality assessment performed for both sequences.							
	**Overall image quality score**				**CMR RV artifact score**		
	**Score 1** **Non-diagnostic**	**Score 2** **Fair**	**Score 3** **Good**	**Score 4** **Excellent**	**Score 0–3**	**Score 4–6**	**Score 7–10**
SSFP_ref_**—***n* (%)	1/61 (1.6%)	10/61 (16.4%)	22/61 (36.1%)	28/61 (45.9%)	47/61 (77.1%)	11/61 (18.0%)	3/61 (4.9%)
CS_rt_**—***n* (%)	0/61 (0.0%)	12/61 (19.7%)	49/61 (80.3%)	0/61 (0.0%)	55/61 (90.2%)	6/61 (9.8%)	0/61 (0.0%)
*p*-value	0.0001				0.0016		
The significance of the Wilcoxon test is defined by *p* < 0.05. Abbreviations: SSFP_ref_, reference steady-state free-precession cine; CS_rt_, real-time compressed-sensing cine; *n (%)*, data represented as numbers (percentages); CMR, cardiac magnetic resonance; RV, right ventricle.							
**b.** Diagnostic performance crosstabulation for tricuspid-regurgitation-flow-related artifact depiction.							
	**SSFP_ref_: TR+**			**SSFP_ref_: TR** **−**	**Total**		
**CS_rt_: TR+**	23/61 (37.7%)			0 (0.0%)	23/61 (37.7%)		
**CS_rt_: TR** **–**	8/61 (13.1%)			30 (49.2%)	38/61 (62.3%)		
**Total**	31/61 (50.8%)			30/61 (49.2%)	61/61 (100.0%)		
Considering SSFP_ref_ as the gold standard, CS_rt_ demonstrated the following diagnostic performances for the depiction of tricuspid-regurgitation-flow-related artifacts: sensitivity = 74.2%; specificity = 100%; positive predictive value = 100%; negative predictive value = 78.9%; area under ROC = 0.87. Abbreviations: SSFP_ref_, reference steady-state free-precession cine; CS_rt_, real-time compressed-sensing cine; TR+, conspicuous tricuspid-regurgitation-flow-related artifact; TR–, no tricuspid-regurgitation-flow-related artifact depicted; ROC, receiver operating characteristic.							

## Data Availability

The data presented in this study are available on reasonable request from the corresponding author, subject to approval by the research ethics committee of Lille University Hospital.

## Supplementary Materials

The following are available online at https://www.mdpi.com/article/10.3390/jcm10091930/s1, Video S1: Four-chamber cine slice in a 31-year-old male patient referred for transposition of great arteries after a Senning repair (same patient as Figure 2); Video S2: Four-chamber cine slice in a 27-year-old female patient referred for tetralogy of Fallot follow-up.
